# A New Multi-Color FISH Assay for Brush Biopsy-Based Detection of Chromosomal Aneuploidy in Oral (Pre)Cancer in Patients with Fanconi Anemia

**DOI:** 10.3390/cancers14143468

**Published:** 2022-07-17

**Authors:** Bruno Eduardo Silva de Araujo, Mona Markgraf, Isabela Karoline de Santana Almeida Araujo, Eunike Velleuer, Ralf Dietrich, Natalia Pomjanski, Martin Schramm

**Affiliations:** 1 Department of Cytopathology, Heinrich Heine University, 40225 Düsseldorf, Germany; mona.markgraf@med.uni-duesseldorf.de (M.M.); isabelakaroline.desantanaalmeidaaraujo@med.uni-duesseldorf.de (I.K.d.S.A.A.); eunike.velleuer@med.uni-duesseldorf.de (E.V.); n.pomjanski@med.uni-duesseldorf.de (N.P.); martin.schramm@med.uni-duesseldorf.de (M.S.); 2 Centre for Child and Adolescent Health, HELIOS Klinikum, 47805 Krefeld, Germany; 3 German Fanconi Anemia Support Group, 59427 Unna, Germany; ralf.dietrich@fanconi.de

**Keywords:** fluorescent in situ hybridization, Fanconi anemia, oral cancer

## Abstract

**Simple Summary:**

Patients with Fanconi Anemia (FA) are at high risk of developing oral squamous cell carcinoma with poor prognosis. Oral brush biopsy–based cytology represents an attractive non-invasive advantage in the characterization of (pre) malignant oral lesions, but a sufficient number of abnormal cells is mandatory to make the diagnosis. The aim of this 2-phases retrospective study is to introduce a new multi-color FISH assay including a 9p21 FISH assay to determine the oral lesions that require treatment, with just a few cells. We demonstrate a good accuracy of the multicolor FISH assay if applied on oral brush biopsy-based specimens from FA patients, using different algorithms to determine chromosomal aneuploidy. Nevertheless, some false positive results were observed and the detection of a genetically altered field in the oral cavity as a possible reason for what may hamper the application of multi-color FISH is discussed.

**Abstract:**

Background: Fanconi anemia (FA) is a rare inherited DNA instability disorder with a remarkably elevated risk of oral squamous cell carcinoma. These cancers can be detected with oral brush biopsy-based cytology even at early stages. This study aims to determine the diagnostic accuracy of a new multi-color fluorescent in situ hybridization (FISH) assay consisting of probes for *CCND1*, *TERC*, *MYC* and centromere of chromosome 6, as well as a 9p21 FISH assay consisting of probes for *CDKN2A* and centromere of chromosome 9 for the detection of oral (pre) malignant lesions in FA. Methods: (I) Cutoffs for the dichotomization of positive or negative multi-color FISH results are determined and (II) retrospectively validated by using archived oral brush biopsy specimens from individuals with Fanconi anemia. In addition, the specimens for cutoff determination were re-hybridized with the 9p21 FISH assay. Results: A cutoff of six or more chromosomal aneuploid cells for a positive FISH result was determined in the cutoff study on 160 biopsy specimens. The validating of this cutoff on 152 specimens showed at best a sensitivity of 87% and a specificity of 82.9%. Conclusion: Multi-color FISH is a sufficient tool to detect chromosomal aneuploidy in oral (pre) malignant lesions of individuals with Fanconi anemia. However, some false positive results may hamper the application as an adjuvant method to oral brush biopsy-based cytology in an oral cancer surveillance program.

## 1. Introduction

Fanconi anemia (FA) is a rare hereditary bone marrow failure and cancer predisposition syndrome caused by germline mutations in any of 22 known genes (*FANCA-W*) which encode proteins of the FA pathway [1,2]. The disruption of the FA pathway is associated with spontaneous and inducible genetic instability, impaired DNA damage repair, compromised mitotic fidelity and sensitivity to interstrand cross-links [1,3,4,5]. These conditions are linked to an increased risk of malignant transformation, mainly hematological malignancies and squamous cell carcinoma (SCC) [3,6]. A 500–700 times higher risk for developing SCCs of the head and neck compared to the general population is estimated for FA patients. The majority of these cancers are located in the oral cavity [7].

Oral squamous cell carcinoma (OSCC) in FA patients is a complex challenge to patients and health professionals, due to its high aggressiveness [8] and limited therapeutic options [7]. To achieve early diagnosis, frequent oral inspections starting at the age of 10 are recommended [9]. Moreover, OSCC in FA is typically pre-ceded by visible oral lesions [10]. The evaluation of those lesions with brush biopsy-based cytology and DNA ploidy analysis accurately identifies oral lesions at risk for malignant transformation [10] and therefore reduces the need of invasive tissue biopsies. In addition, benign oral lesions, which are very prevalent in FA, and which do not require invasive diagnostic work-up, can be precisely differentiated from (pre) malignant lesions [10]. However, due to inconclusive (atypical or suspicious) cytology or an insufficient number of suspicious cells (<100 cells) for an additional DNA ploidy analysis with DNA image cytometry, some lesions cannot be classified with oral brush biopsy-based cytology alone and still require tissue biopsies.

Fluorescent in situ hybridization (FISH) is a technique that could overcome this limitation, due to its high accuracy, to detect early cytogenetic changes in cytological specimens [11] even with a relatively low number of suspicious cells (i.e., 25–50 cells). Moreover, the detection of chromosomal aneuploidy with multi-color FISH is commonly used in diagnostic cytopathology to overcome limitations of inconclusive diagnostic results [11,12]. Data from a proof-of-concept study conducted by our workgroup [13] showed that all of the studied FISH probes showed copy number variations in most of the FA-related OSCC cell lines and oral brush biopsy-based smears from (pre) malignant lesions. From the 13 FISH probes studied, 4 commercially available probes *CCND1* (*Cyclin D1*), *TERC* (*Telomerase RNA Component*), *MYC* (*MYC proto-oncogene*) and CEP6 (centromere chromosome 6) labelled with different fluorochromes showed the potential to be combined to a multi-color FISH assay to detect chromosomal aneuploidy. In addition, a relative 9p21 deletion was detected in most of the OSCC specimens, indicating a potential use in a diagnostic FISH assay combined with a corresponding centromeric probe of chromosome 9.

This present study aims to determine the diagnostic accuracy of a new multi-color and a 9p21 FISH assay for the detection of chromosomal aneuploidy in oral (pre) malignant lesions in FA. Cutoffs for the dichotomization of positive or negative FISH results are determined and retrospectively validated by using archived oral brush biopsy specimens from FA individuals, respectively.

## 2. Materials and Methods

### 2.1. Study Design and Samples

All samples were provided by FA individuals that participated in an oral cancer surveillance study from 2007 to 2017 and were processed as described by Velleuer et al., 2020 [10]. Oral lesions were sampled using cervical or oral brushes. Exfoliated cells were processed for liquid-based cytology or smeared on a microscopic slide and immediately fixed with alcohol spray (Merckofix; Merck, Darmstadt, Germany) for conventional cytology. The cytopathological diagnosis was performed at the cytopathology department of the Heinrich Heine University in Düsseldorf, Germany. Residual archived Papanicolaou-stained oral smears and liquid-based preparations from this previous study were used in our FISH study.

The present FISH study was carried out in accordance with the 1964 Helsinki declaration and its later amendments and was approved by the ethics committee of the medical faculty of the Heinrich Heine University in Düsseldorf, Germany (study number 2019-625). All FA individuals provided written informed consent to donate the samples for future research and/or methods development.

**Figure 1:**
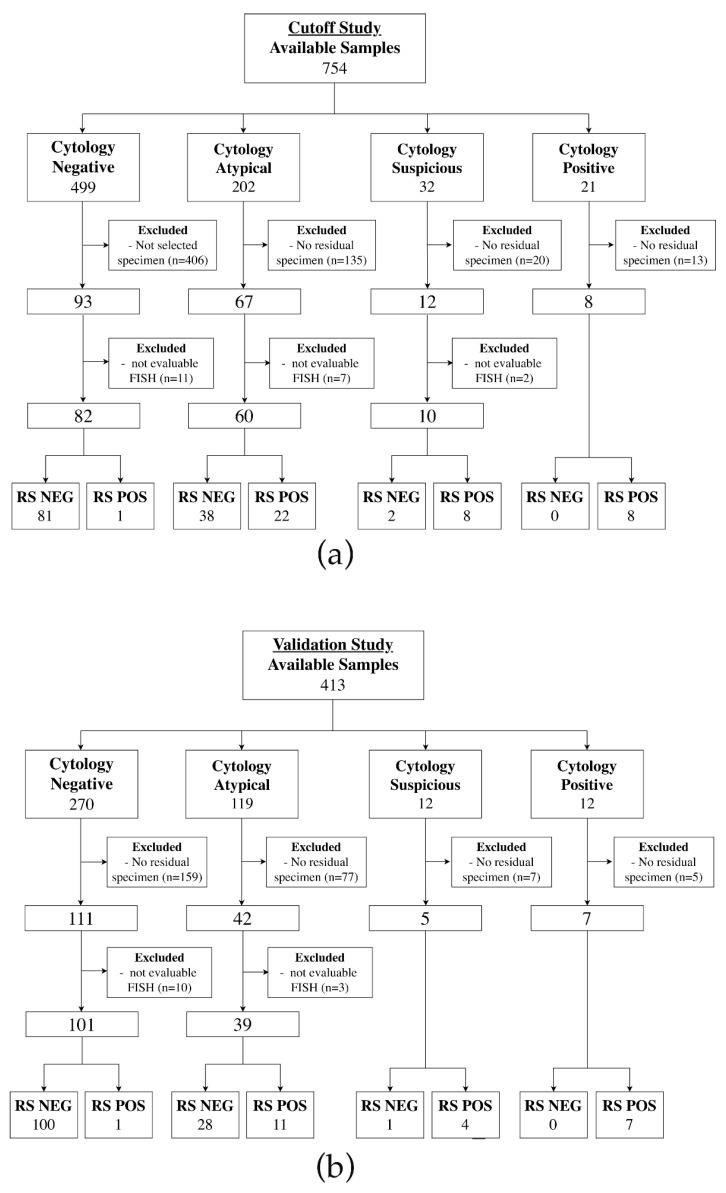

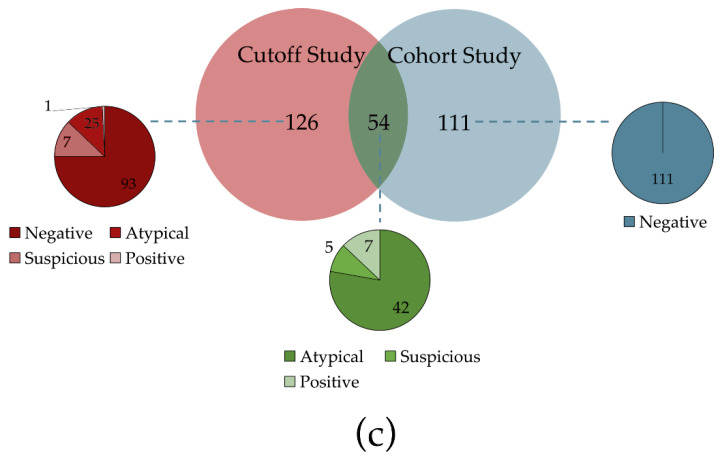
Flow diagrams and modified Venn diagram demonstrating sample inclusion, dropouts for FISH analysis, the reference standard and overlapping relationship between study phases. (**a**) Flow diagram demonstrating the cutoff study. All well preserved samples, stained according to Papanicolaou with an available reference standard, were considered for inclusion in the cutoff study. (**b**) Flow diagram of the validation study. The cohort was limited to the period of 2014 to 2017. (**c**) Modified Venn diagram demonstrating the number and cytological diagnoses of samples that were included to exclusively one phase of the study (red or blue) or both phases (green). RS NEG, negative clinicopathological follow-up reference standard; RS POS, positive clinicopathological follow-up reference standard.

Based on microscopic re-evaluation, archived samples that had enough well preserved normal epithelial cells or abnormal cells as described in paragraph 2.2 were selected. In general, for each brushed lesion there was more than one archived sample available but not all samples contained the relevant cells corresponding to the final cytological diagnosis. Accordingly, each sample was microscopically re-evaluated before inclusion in the study. Many oral lesions from the oral cancer surveillance study were excluded because appropriate residual samples were missing (Figure 1).

The FISH study was divided in two parts defined as cutoff study and validation study. All FISH analyses were performed blinded to the follow-up data.

#### 2.1.1. Cutoff Study

In the cutoff study, 93 negative cytological samples and 87 samples cytologically classified as atypical, suspicious and positive were included (Figure 1a). The samples were selected based on all 754 archived oral smears obtained during 2007 to 2017 from an oral cancer surveillance study with available follow-up data [10].

#### 2.1.2. Validation Study

Cytological samples for the validation study have been chosen out of the cohort between 2014 to 2017 as it concentrated the highest number of eligible and best-preserved samples. The selected cutoff obtained from the cutoff study was applied to 165 samples out of 413 oral smears of which 111 samples have been cytologically negative, 42 atypical, 5 suspicious and 7 positive (Figure 1b). All atypical, suspicious and positive samples were already analyzed in the cutoff study and the results with the appropriate cutoff were included in the validation study (Figure 1c). This approach was necessary due to a low amount of eligible cytologically samples. The negative samples from the cutoff study were not included in the validation study.

### 2.2. Re-Evaluation of Cytological Samples

Papanicolaou-stained conventional smears or cytospins from liquid-based cytology were microscopically re-evaluated by experienced cytopathologists (coauthors N.P. and/or M.S.) in order to define the areas on a sample with the highest number of eligible normal or abnormal cells. The borders of those areas were scratched with a diamond pen.

The diagnostic categories for cytology according to Velleuer et al., 2020 [10] were: “negative” indicating normal squamous cells and reactive or inflammatory changes; “atypical” indicating atypical cells present (e.g., superficial cell dyskaryosis); “suspicious” indicating dyskaryotic cells of the parabasal type or only a few malignant cells present; “positive” indicating malignant cells present.

### 2.3. FISH

Probes used for the multi-color FISH assay were *CCND1* (Vysis LSI *CCND1*, SpectrumGreen), *TERC* (Vysis LSI *TERC*, SpectrumGold), *MYC* (Vysis LSI *MYC*, SpectrumRed) and centromere of chromosome 6 (Vysis CEP 6, SpectrumAqua).

The 9p21 FISH probe consisted of a probe for *CDKN2A* (Vysis LSI *CDKN2A*, SpectrumOrange) and the corresponding centromeric probe of chromosome 9 (Vysis CEP 9, SpectrumGreen). All FISH probes were obtained from Abbott (Abbott Molecular Inc., Des Plaines, IL, USA).

#### 2.3.1. Hybridization

In each sample, the multi-color FISH was applied according to the recommendations of the manufacturer with some modifications. The cytological slides were uncovered in xylene for 2 days to remove the coverslip and placed in another coplin jar with fresh xylene for 3 days to remove possible remnants of the mounting medium. The samples were rehydrated twice for 5 min each in 100%, 95% and 80% ethanol and transferred to 0.5% HCl/70% ethanol for 15 min at room temperature. Afterwards, the slides were carefully washed under running tap water for 5 min, immersed in 2X saline sodium citrate (SSC) for 5 min at 73 °C and digested by using 0.2 mg/mL pepsin in 0.01 mol/L HCl for 15 min at 37 °C in a humidified chamber. The slides were washed in 10% phosphate-buffered saline (PBS) for 5 min at room temperature, fixed in 1% neutral-buffered formalin/PBS for 5 min and washed in PBS again for 5 min, subsequently. After dehydration for 2 min each in 70%, 85% and 100% ethanol the slides were allowed to air dry at room temperature.

The respective FISH probes for the multi-color assay and the 9p21 assay were mixed with Vysis IntelliFISH Hybridization Buffer (Abbott Molecular Inc., Des Plaines, IL, USA) and purified water resulting in a 20% solution. Accordingly, 6 µL probe mix for the multi-color FISH consists of 0.3 µL of each FISH probe, 4.2 µL buffer and purified water. For the hybridization of a 15 mm × 15 mm area on a sample, 6 µL of the probe mix was used. If necessary, differently sized areas were selected for hybridization and the amount of the probe mix was adjusted. The probe mix was applied on a fresh coverslip with a pipette and the cytological slide was pressed against this coverslip in the region marked with a diamond pen. Thermal glue sealed the edge of the coverslip to prevent air drying and the samples were placed for 5 min in a humidified chamber at 37 °C.

After denaturation at 73 °C for 10 min, the slides were incubated at 37 °C overnight in a humidified chamber for hybridization. After hybridization, the samples were incubated for 2 min in 0.4X SSC/0.1% nonyl phenoxypolyethoxylethanol (NP-40) at room temperature to remove the coverslips, washed again for 2 min in 0.4X SSC/0.1% NP-40 heated at 73 °C in a water bath and subsequently for 1 min in 0.4X SSC/0.1% NP-40 at room temperature. Then, the slides were dipped 5 times in distilled water, air dried and covered with 2 drops of 4.6-diamidine-2-2phenylindole dihydrochloride (DAPI) (Abbott Molecular Inc., Des Plaines, IL, USA) for counterstaining. Finally, a fresh coverslip was placed and the edges were sealed with thermal glue.

#### 2.3.2. Re-Hybridization

All samples of the cutoff study that were successfully analyzed with the multi-color assay were unstained and re-hybridized with the 9p21 FISH assay.

To remove the coverslips, the slides were washed in 100% ethanol for 5 min and air dried at room temperature. After 5 min in 0.4X SSC/0.1% NP-40 at room temperature to remove DAPI, the slides were immersed in 0.4X SSC/0.1% NP-40 heated at 85 °C in a water bath and then immediately in 2X SSC at room temperature for 3 min each. In another coplin jar with fresh 0.4X SSC/0.1% NP-40 at 85 °C the removal of the FISH probes was finalized. The slides were washed in 2X saline sodium citrate (SSC) at room temperature for 3 min and placed in distilled water at room temperature until the water bath cooled down to 73 °C (circa 20 min).

The samples were immersed in 2X saline sodium citrate (SSC) for 5 min at 73 °C, cleansed in 70%, 85% and 100% ethanol for 2 min each and allowed to air dry at room temperature.

The *CDKN2A* probe and the CEP 9 probe for the 9p21 assay was readily provided by the manufacturer. For a 15 mm area, 0.6 µL of *CDKN2A*/CEP9 probes was mixed with 4.2 µL buffer and 1.2 µL purified water.

#### 2.3.3. FISH Analysis

The hybridized areas on the specimens were analyzed for atypical nuclei (nuclear enlargement, irregular shape, patchy DAPI staining) using an Axio Imager M1 microscope (Carl Zeiss Microscopy, Jena, Germany) equipped with ×63 and ×100 oil immersion objective lenses. Up to 50 nuclei of interest for each specimen were analyzed by two independent observers in duplicates (coauthors B.E.S.d.A. and M.M.) and the number of signals for each probe was registered. The quality hybridization in neutrophils were used as an internal reference. Diffuse and split signals were counted as one signal. The distance between signals to be considered unique was at least two signal widths. It was avoided to analyze ill-defined or overlapping nuclei, nuclei with artefacts due to bacteria or cells with an intense cytoplasmatic or background fluorescence.

Microphotos of DAPI-stained nuclei and fluorescent signals were taken with a Zeiss AxioCam MRm 1.4 Megapixel camera (Carl Zeiss Microscopy, Jena, Germany), and edited with the AxioVision SE64 Rel. 4.9 Software (Carl Zeiss Microscopy Deutschland, Oberkochen, Germany).

#### 2.3.4. FISH Evaluation Protocol

Chromosomal aneuploidy was measured with the multi-color FISH assay whereas homozygous and relative deletion have been analyzed using the 9p21 FISH assay.

For the detection of chromosomal aneuploidy, 4 different algorithms were defined. The aim was to evaluate the best diagnostic accuracy for the detection of at least a high-grade oral epithelial dysplasia. A cell was considered chromosomally aneuploid if:

- At least 3 FISH probes showed a gain (>2 signals) (definition 1 (D1));
- All 4 probes showed a gain (>2 signals) (definition 2 (D2));
- All 4 probes showed a gain (>2 signals) or 3 probes showed a gain (>2 signals) if one of these probes was MYC with a gain of ≥4 Signals (definition 3 (D3));
- All 4 probes showed a gain (>2 signals) or 3 probes showed a gain (>2 signals) if one of these probes was MYC with a gain of ≥5 Signals (definition 4 (D4)).

Tetrasomy was not considered chromosomally aneuploid since it occurs both in malignant tumors and in physiological euploid polyploidization. Tetrasomy was defined as the presence of four copies of the genes in a nucleus. A deviation of ±1 copy of one of the genes, as previously suggested [12], was accepted. Thus, the following patterns of gene copies were accepted as tetrasomy, regardless of the order: 4-4-4-4, 5-4-4-4, 3-4-4-4 [13].

**Figure 2:**
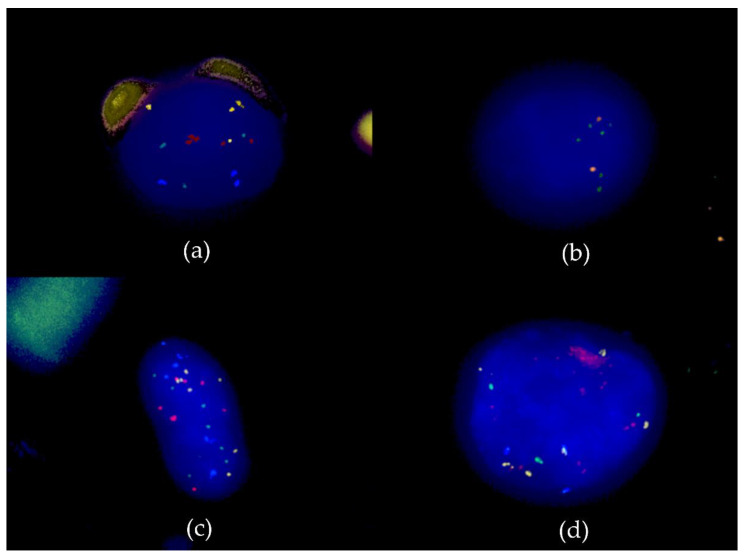
Representative images of the multi-color fluorescent in situ hybridization (FISH) assay (*CCND1*, green; *TERC*, yellow; *MYC*, red; CEP6, blue) and the 9p21 FISH assay (CEP9, green; *CDK2NA*, orange). (**a**) Chromosomal aneuploidy with 3-4-4-3 signals for *CCND1*, *TERC*, *MYC* and CEP6, respectively (100× magnification). (**b**) Relative deletion of 9p21 (CEP9, green; *CDK2NA*, orange) (100× magnification). (**c**) Image of 2 cells with normal morphology in DAPI stain but showing a high number of FISH signals (63× magnification). (**d**) Chromosomal aneuploidy and cluster of *MYC* signals (definition of chromosomal aneuploidy D4) (100× magnification).

Regarding the analysis of the *CDKN2A* and CEP9 probes for each slide, 2 definitions were created to identify homozygous deletion (HoD) and relative deletion (ReD) of 9p21, respectively. A relative deletion of 9p21 in a given nucleus was observed if the number of the *CDKN2A* signals was lower than the number of CEP9 signals. A homozygous deletion was defined as the absence of *CDKN2A* signals in nuclei with at least 1 centromere 9 signal [14]. Representative images of the multi-color FISH assay and the 9p21 FISH assay are shown in Figure 2.

### 2.4. Reference Standard

The results of the reference standard refer to the location and the time of the respective brush-biopsies from the visible oral lesions and were according to Velleuer et al., 2020 [10]. A positive reference standard for a distinct oral lesion was defined as: (I) a histological diagnosis of OSCC or high-grade oral epithelial dysplasia (OED) (including moderate and severe OED) [15] within 6 months of oral examination or (II) a positive cytological diagnosis or the detection of DNA aneuploidy with a consistent clinical course (i.e., OSCC therapy, definite imaging or palliative care). The negative reference standard was defined as either a negative (benign) or low-grade OED histological diagnosis within 6 months or a negative clinical course within 2 years of oral examination.

### 2.5. Statistical Analysis

Statistics were performed using the software IBM SPSS Statistics (Version 25; IBM Corporation, New York, NY, USA) (coauthor I.K.d.S.A.A.). In the cutoff study, 5 different codes were created in SPSS syntax editor, 1 to identify tetraploid cells, and the other 4 to identify aneuploid cells, each one with different definitions (D1, D2, D3 and D4). These codes were applied to the numbers of FISH signals in each analysis, with the tetraploid code being applied first, followed by the other 4 codes (D1, D2, D3, D4). Those datasets describe the diagnostic accuracy if either the FISH results of all specimens or only the ones of the atypical, suspicious and cytologically positive specimens were calculated.

The receiver operating characteristic (ROC) curve was used to compare the classification efficiency of D1, D2, D3 and D4, as well as HoD and ReD.

For the dichotomization of the data, the sensitivity and specificity for the application of each of the definitions of aneuploidy (D1 to D4) was calculated at different cutoffs of aneuploid cells. The optimal cutoff was calculated using the Youden Index [16]. Regarding the comparison of the 4 definitions, their respective sensitivity and specificity values were taken into account, as well as their respective area under a ROC curve (AUC) of the chosen point. Cohen’s kappa [17] was applied to analyze the agreement and reliability between the independent observers (coauthors B.E.S.d.A. and M.M.). The level of significance was set to (α) = 0.05.

For the validation study, the same codes were applied. Finally, the sensitivity, specificity and AUC values for the cutoff point defined with the cutoff study were calculated.

## 3. Results

### 3.1. Patients and Samples

In the cutoff study, out of the 180 samples, a total of 160 samples could be analyzed with the multi-color FISH assay (Figure 1a). These samples were provided by 80 patients, who contributed 1–13 samples. The 9p21 FISH assay following re-hybridization could be applied to 154 of the samples. The six samples, which could not be evaluated due to weak fluorescent signals, all had an atypical cytology.

Out of 165 samples, 152 could be analyzed in the validation study (Figure 1b). They originated from 77 patients who contributed 1–10 samples.

Detailed characteristics of the patients and of the cytological diagnoses for both parts of the study are reported in Table 1 and Figure 1.

In the cutoff study, 24.4% (39 out of 160) of the samples had a positive follow-up reference standard. Of these 39 samples 22, 8 and 8 had an atypical, suspicious and positive cytological diagnosis, respectively. This represents 36.7% of all atypical, 80% of all suspicious and 100% of all cytologically positive samples (Figure 1a).

In the validation study, 15% (23 out of 152) of the samples included had a positive follow-up reference standard. The share of cytologically atypical, suspicious and positive samples with a positive follow-up is 28.2%, 80% and 100%, respectively (Figure 1b).

### 3.2. Multi-Color FISH Assay

After a thorough analysis of the diagnostic accuracy for each definition of chromosomal aneuploidy (D1 to D4) (Appendix A—Table A1 and Table A2), a cutoff of six or more aneuploid cells for a positive FISH result was selected based on the highest Youden indices for definition D4 in the respective analyses.

The calculation of agreement and reliability between the independent observers was performed for each kind of analyses (D1–D4; all specimens vs. atypical cytology only). The kappa value ranged from 0.846 (D1 for all specimens) to 0.476 (D1 for atypical cytology only). The level of agreement ranged from moderate to perfect.

The diagnostic accuracy was first calculated for all samples to determine false positive and false negative rates, as well as specificity and sensitivity. Moreover, a second calculation just for the atypical, suspicious and positive samples was performed, because it is expected that the use of the FISH technology is better suited for a characterization of the latter ones in a multimodal approach.

Table 2 displays all the results of the multi-color FISH assay using a cutoff of six or more aneuploid cells. For the cutoff study, all definitions of chromosomal aneuploidy were analyzed, whereas for the validation study just the definitions D2 and D4 were included due to the best results in the cutoff study.

The best results with higher Youden indices were found for the evaluation of all samples, including the ones with negative cytology, either in the cutoff study or in the validation study. The specificity was higher, compared to the analyses for atypical, suspicious and positive cytological samples only, as expected, because most cytologically negative samples had a negative FISH result. In the cutoff study, D1 showed the best sensitivity of 89.7%, but the specificity of 72.7% is too low for usage as a diagnostic test to aid or confirm the cytological diagnosis.

In the cutoff study, D2 and D4 showed the highest specificities of 86% and 83.5%, respectively, and hence were chosen as definitions of chromosomal aneuploidy for the validation study. Compared to the cutoff study, the specificity values of both definitions were slightly decreased in the validation study (Table 2), mainly caused by some false positive FISH results of cytologically negative samples (Table 3).

Regardless of the algorithm used to define a cell as chromosomal aneuploid, some samples were classified as false positive regardless the applied definition. This finding may indicate genetic changes in the analyzed chromosomal regions that were not related to a malignant transformation within the limits of the chosen follow-up periods. To investigate other hypotheses, the relation of FISH results and either cytological diagnostic categories or information regarding a human stem cell transplantation (HSCT) in the clinical history was analyzed using the definition D2 of chromosomal aneuploidy and a cutoff value of six or more aneuploid cells for dichotomization (Table 3). In total, 24 lesions from 21 patients were classified as false positives in the cutoff study and validation study by using the definition D2. Of these lesions, 13 were included in both parts of the study. Three patients each had two oral lesions classified as false positives.

In the cutoff study, atypical cytology samples were responsible for 94% of the false positive and 87.5% of the false negative FISH results (Table 3). However, 37 out of 60 (61.7%) cytologically atypical samples were true positives or negatives. Only one cytologically negative sample each was false positive or false negative and all cytologically suspicious and positive samples had true positive or true negative FISH results.

In the validation study, atypical cytology samples were responsible for 65% of the false positive and 100% of the false negative FISH results (Table 3). However, in contrast to the cutoff study, 35% of the false positive FISH results were observed in the cytologically negative samples. Again, all cytologically suspicious and positive samples had true positive or true negative FISH results. No correlation of a HSCT in clinical history and the results of multi-color FISH, whether in the cutoff or in the validation study, was observed.

In order to find possible reasons for the high rate of false positive FISH results using the definition D2, a prolonged and enhanced follow-up was obtained from those patients (Appendix B—Table A3). It was analyzed whether those lesions correspond to a high-grade OED or a SCC at any place in the oral cavity or even in the neighborhood of the false positive FISH lesions, either in the clinical history, at the same time of brushing or in an enhanced follow-up period. For 10 of the 24 false positive lesions, no high-grade OED or SCC was reported. Five lesions were brushed from FA patients that had oral high-grade OED or SCC either in the clinical history or in the enhanced follow-up period, but not in the vicinity of the lesion with false positive FISH results. In addition, nine lesions, oral high-grade OED or SCC either in the clinical history or in the enhanced follow-up period, occurred in the vicinity or the same place of the lesion with false positive FISH results (Appendix B—Table A3).

### 3.3. 9p21 FISH Assay

**Figure 3:**
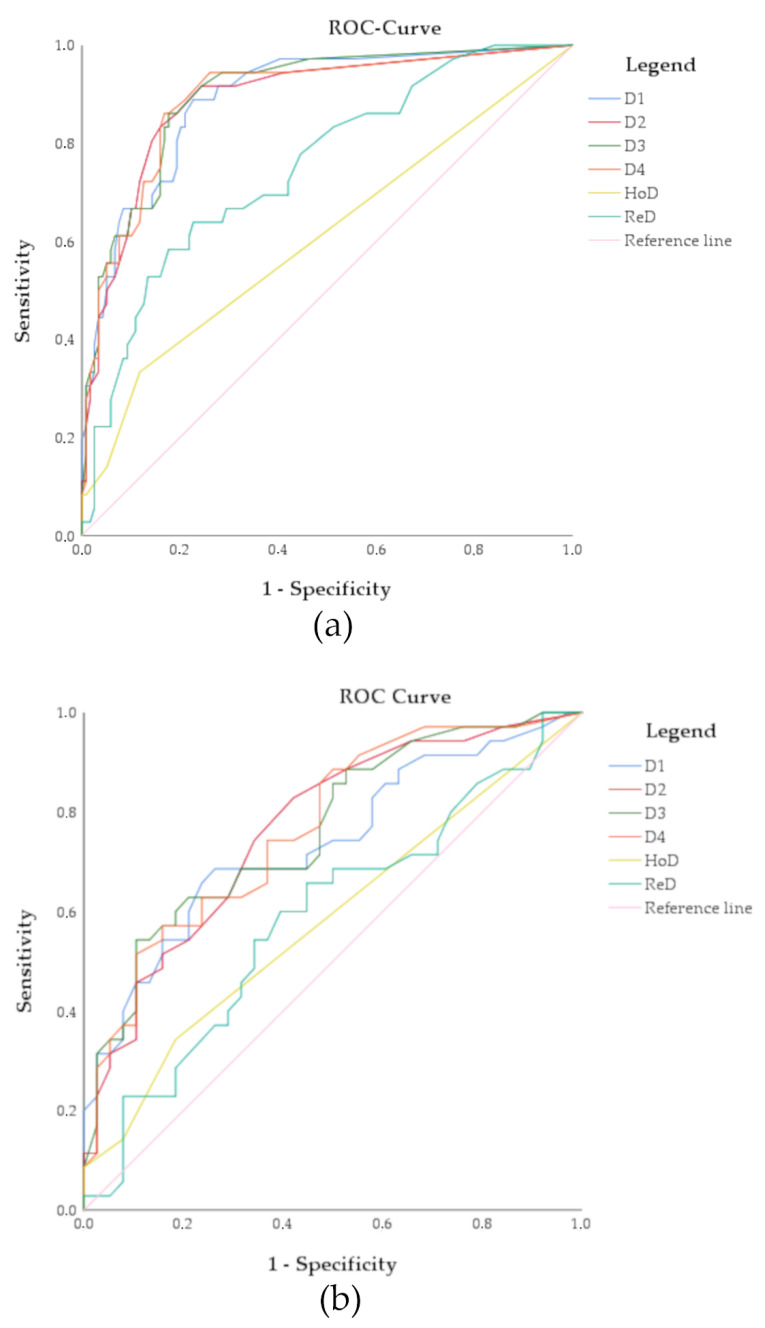
ROC curves of the multi-color FISH assay and the 9p21 FISH assay. (**a**) Results for all specimens; (**b**) Results just for the cytologically atypical, suspicious and positive samples. Abbreviations: D1–4, definitions of chromosomal aneuploidy D1 to D4; HoD, homozygous deletion of 9p21; ReD, relative deletion of 9p21.

Only the samples from the cutoff study were analyzed with the 9p21 FISH assay subsequent to the multi-color FISH assay after re-hybridization. The ROC curve analysis showed that a homozygous deletion of 9p21 could not predict the clinic-histological outcome, because it is close to the reference line (Figure 3). Even the analysis of a relative deletion of 9p21, already present in many of the cytologically negative samples, could not sufficiently differentiate lesions with a negative or positive follow-up (Table 4).

## 4. Discussion

In an effort to aid the brush biopsy-based early diagnosis of oral cancer in visible lesions from FA individuals [10], this study aimed to establish a multi-color FISH assay that detects chromosomal aneuploidy. After demonstrating that FISH shows different signal patterns for cancerous and benign cultured or exfoliated cells derived from oral lesions in a proof of concept study [13], four FISH probes covering *CCND1*, *TERC*, *MYC* and the centromere of chromosome 6 (CEP6) with different fluorochromes were combined to a multi-color assay. In addition, a 9p21 FISH assay was analyzed, because a 9p21 deletion was reported for (pre) malignant oral lesions in the general population [18]. Moreover, the loss of heterozygosity and a relative deletion of 9p21 was detected in cells derived from oral lesions of FA individuals [13,19]. A clinical cutoff for the detection of chromosomal aneuploidy (=FISH positive) was determined (cutoff study) and validated (validation study). Multi-color FISH to detect chromosomal aneuploidy in cytological specimens was already applied to other organs [11,20] but was not systematically analyzed in oral lesions of individuals with FA.

FA patients who have undergone a hematopoietic stem cell transplantation (HSCT) are at an even higher risk of developing oral cancer than those without HSCT [21]. Table 1 shows that approximately two-thirds of lesions in both parts of the study were provided by transplanted patients. This may represent a potential bias towards higher numbers of malignant oral lesions. However, the samples for the FISH analyses were not selected based on the HSCT status and the share of transplanted FA patients with positive follow-up is lower than the share of non-transplanted FA patients with positive follow-up. Most of the transplanted patients had a negative reference standard in the cutoff study as well as in the validation study (Table 1).

One big challenge of applying FISH for an early diagnosis of cancer is the ability to differentiate genetic alterations linked to benign processes such as inflammation, infection and regeneration from those alterations occurring in (pre-) cancerous lesions. Regenerating or inflammatory processes often show euploid polyploidization that has 2^n^ chromosomal sets (e.g., tetrasomy) [22] and that should be considered in scoring algorithms for a multi-color FISH assay [12]. Reflecting this, we tested several diagnostic algorithms (D1–D4) for the detection of chromosomal aneuploidy on a series of 160 evaluable brush biopsy-based samples from oral lesions of FA individuals. A cutoff of six or more chromosomally aneuploid cells was determined for a positive FISH result that is useful in the diagnostic routine and in line with other multi-color FISH applications [12]. The algorithms D2 (gain >2 signals in all four FISH probes) and D4 (gain >2 signals in all four probes or at least three probes showed a gain if one of these probes was *MYC* with ≥5 signals) showed the best outcomes in the cutoff study (Table 2). Many samples with a negative follow-up presented a high number of cells with three probes showing at least three signals and only one probe without gain. Thus, a higher number of probes with a gain or a higher number of *MYC* fluorescent signals seemed to be necessary to specifically detect lesions with a high risk of malignancy. Some studies have already demonstrated a higher rate of *MYC* gain, polysomy or amplification in OSCC or oral lichen planus that progressed to OSCC than in normal mucosa or lesions that did not progress [23,24]. For validation, the established cutoff was applied to a series of 152 evaluable brush biopsy-based samples from oral lesions of FA individuals with comparable diagnostic accuracy for the aneuploidy definitions D2 and D4.

Nevertheless, even after application of very stringent definitions of chromosomal aneuploidy compared to the literature [25,26] and considering cells with a tetraploid pattern as non-aneuploid, we observed a high number of samples with a false positive FISH result either in the cutoff study or in the validation study (Table 3). The samples with a false positive FISH diagnosis usually had a prior cytologically atypical or sometimes a negative diagnosis, whereas suspicious or positive cytology were always true negatives or true positives with multi-color FISH. This high number of probe signals in lesions with a negative follow-up within the limits of our definition of the reference standard may probably be explained by a genetic field defect and/or difficulties in signal counting due to degraded DNA.

According to the field defect theory (also known as field effect in cancer or field cancerization), benign tissue with microscopically normal, hyperplastic or atypical epithelium that is in proximity to tumors, has some similar genetic alterations compared to the cells in the fully developed tumors [27,28]. FA patients usually present some clinical features linked to a field defect as subsequent tumor development within or in proximity to a genetically altered field, synchronous or metachronous primary tumors and local recurrences [29,30]. Furthermore, cancer stem cells as part of a field defect give rise to new tumors [31] and were already reported in FA-OSCC [32]. As described in the subSection 3.2, nine of the lesions with a false positive FISH result had a positive extended follow-up in the same oral region or were located in the vicinity of a region that already had or developed high-grade OED or SCC. Five additional false positive lesions with FISH were from patients that had high-grade OED or SCC either in the clinical history or in the enhanced follow-up period elsewhere in the mouth but not in the vicinity. Thus, as a highly sensitive method, FISH may detect early genetic alterations that are associated to a genetically altered field prior to a malignant transformation or to a genetically altered field in a temporal or geographical context to a high-grade OED or OSCC.

Liquid-based cytology clearly demonstrated weaker fluorescent signals than conventional smears in tests of the FISH methodology that were performed prior to our FISH studies (unpublished data). The analysis of liquid-based samples was only possible after adjusting the FISH staining protocol by increasing the pre-treatment time in Xylene from 2 to 72 h. Moreover, the cells in liquid-based preparations frequently showed split signals that sometimes hampered the analysis. Kim et al. [33] showed accelerated DNA degradation in archived liquid-based cytology in comparison to conventional smears, using aspirates from thyroidectomy specimens.

Considering just the results of the multi-color FISH assay with algorithm D4 with a cutoff of 6 or more chromosomally aneuploid cells, 34 of 39 (87.2%) samples with a positive follow-up were FISH-positive in the cutoff study. In the validation study, 20 of 23 (87.0%) samples with a positive follow-up were FISH-positive. This demonstrates the good overall sensitivity of multi-color FISH, especially if compared to the microscopic cytological diagnosis. In the largest cohort analyzing oral lesions from patients with FA [10], atypical and suspicious cytology had a positive reference standard in just 33.6% (48 of 143) and 76% (19 of 25) of the cases with atypical and suspicious cytology, respectively. Thus, a positive FISH result may have a greater impact for the decision of surgical treatment or further observation of a given oral lesion than atypical (or suspicious) cytology alone.

At the time, when we selected possible FISH probes for a multi-color FISH assay, less was known about cytogenetic aberrations in oral SCCs from FA patients and we selected eligible FISH probes according to the available body of literature for the common population regarding FISH analyses in oral (pre) malignant lesions. However, since, a large-scale genomic analysis on FA-related SCC Webster et al. has shown copy number variations in several genetic loci that were covered by the FISH probes we used for our study. Among the amplified loci were *MYC* (69%) and *CCND1* (62%) [34]. The application of this FISH protocol in oral lesions of non-FA patients may be considered, because initially we selected promising FISH probes based on the literature of sporadic (pre) malignant oral lesions. The cancer genome Atlas network provided comprehensive data on structural alterations in SCC of the head and neck [35]. Most tumors show high chromosomal instability with common copy number alterations (CPA) including amplification of 3q. Recurrent amplification of 3q36/28 occur both in human papilloma virus (HPV)-negative and -positive tumors. This region contains the *TERC* gene, that is included in our multi-color FISH panel, and the oncogene *PIK3CA* (phosphatidylinositol 3-kinase, catalytic subunit alpha). In addition, HPV-negative tumors often show amplification of 11q13 including *CCND1* and focal deletions of tumor suppressor genes including *CDKN2A*. According to this data, our multicolor panel may be promising but some modifications may be necessary, i.e., replacing the *TERC* probe with a *PIK3CA* probe and including the *CDKN2A* probe. HPV infection is not a main etiologic factor for FA oral SCC [34] but for the common population, especially oropharyngeal SCC. In HPV-positive SCC of the head and neck 3q CPA, *PIK3CA* mutations and chromosome 16q losses are frequent [36]. Especially the latter genomic regions that are not covered by the FISH probes selected in our study, that may affect the diagnostic accuracy of our multi-color FISH panel to those tumors. In addition, the recently characterized subgroup of CPA silent HPV-negative SCC of the head and neck may be missed by our test that is intended to detect chromosomal aneuploidy [36].

Analysis of 9p21 deletion was applied in our study in order to complement the analysis with the multi-color FISH assay. A significant homozygous deletion of 9p21 was not observed in the samples of the cutoff study, which were re-hybridized and analyzed with the 9p21 FISH assay. In addition, the rate of cells with relative deletion of 9p21 in the cytologically negative samples with negative follow-up was so high that it was decided to limit the analysis to the cutoff study. The determined cutoff for a relative 9p21 deletion of 16 (Table 4) or more cells cell in a sample according to the highest Youden index may not be clinically applicable for the diagnostic routine. The determined sensitivity and specificity were too low and the corresponding ROC curve (ReD) was mostly lower than the ROC curves for the different analyses of chromosomal aneuploidy with the multi-color FISH assay (Figure 3). Therefore, the 9p21 assay would not add any diagnostic benefit to the multi-color FISH assay. These results were unexpected, because in our previous FISH pilot study, five out of six FA-derived OSCC specimens displayed a relative deletion of 9p21 [13]. In addition, recently published data from sequenced FA SCC show a deletion of *CDKN2A* in 55% of samples [34]. However, these studies did not analyze the 9p21 deletion in non-cancerous cells.

As an expensive and time-consuming test, FISH would hardly be applied in the diagnostic routine as an exploratory test. Thus, we additionally calculated the diagnostic accuracy of the multi-color FISH assay of just the cytologically atypical, suspicious and positive samples. Table 2 shows that this approach reduced the specificity compared to the analyses with all samples including the cytologically negative ones. This is, to a great extent, caused by false positive multi-color FISH results in the cytologically atypical and, to a lesser extent, in the cytologically negative samples compared to the reference standard of the study. However, as discussed above, these false positive results could be at least in part attributed to genetically altered mucosal fields and possibly represent early diagnoses or indicate recurrence rather than false positive diagnoses. Nevertheless, the problem of DNA degradation in archived liquid-based specimens should be excluded from future prospective FISH studies on oral (pre) cancerous lesions in FA. Moreover, FISH remains the only technique capable of detecting genetic alterations requiring few abnormal cells and without leukocyte contamination, that is based on cellular morphology.

## 5. Conclusions

The multi-color FISH assay demonstrated to be a sensitive diagnostic method to clarify atypical, suspicious and positive cytology in FA patients demanding only few abnormal cells. FISH could add value to the established diagnostic procedure using brush biopsy-based oral cytology and DNA ploidy analysis [10], albeit on the cost of reduced specificity. One possible reason for the reduced specificity may be the presence of a genetic mucosal field defect and cancer stem cells in lesions that are not defined as (pre) malignant according to the applied reference standard. Since as sometimes there are genetic similarities between the cells of a tumor and benign adjacent tissue that tends to progress, multi-color FISH may have a higher specificity if a prolonged follow-up is applied.

To test the impact of DNA degradation in archived liquid-based cytological specimens, multi-color FISH on oral brush biopsy-based cytology in FA should finally be analyzed prospectively on fresh samples.

## Figures and Tables

**Table 1:** Clinical and demographic characteristics of the individuals with FA included in the cutoff study and the validation study. The data were reported for each oral lesion.

Cutoff Study						
Characteristics	All		No HSCT		HSCT	
	Negative FUP	Positive FUP	Negative FUP	Positive FUP	Negative FUP	Positive FUP
Total No.	121	39	38	15	83	24
Sex, F/M	56/65	13/26	20/18	11/4	36/47	2/22
Median age at brush cytology (Range), Y	26 (10–46)	30 (9–40)	33 (10–46)	33 (9–40)	25 (10–41)	29 (16–36)
Median follow-up time (Range), Mo	45 (9–86)	31 (0–101)	35 (9–66)	9 (0–55)	48 (14–86)	58 (7–101)
Validation Study						
Characteristics	All		no HSCT		HSCT	
	Negative FUP	Positive FUP	Negative FUP	Positive FUP	Negative FUP	Positive FUP
Total No.	129	23	39	9	90	14
Sex, F/M	60/69	6/17	21/18	4/5	39/51	2/12
Median age at brush cytology (Range), Y	27 (10–46)	30 (16–36)	32 (10–46)	30 (16–35)	26 (10–41)	22 (16–36)
Median follow-up time (Range), Mo	33 (7–99)	16 (7–58)	32 (9–59)	9 (7–31)	36 (7–99)	20 (7–58)

Abbreviations: FUP, follow-up; HSCT, hematopoietic stem cell transplantation; No., number; M, male; F, female; Y, years; Mo, months.

**Table 2:** Results of the multi-color FISH assay for various definitions of chromosomal aneuploidy, using a cutoff of 6 aneuploid cells. The results are reported based on calculations for all samples and in addition for those samples with atypical, suspicious and positive cytology only.

		Definition	Sensitivity	Specificity	Youden Index	AUC	AUC *p*-Value	AUC 5% Confidence Interval
Cutoff Study	All specimens	D1	89.7% (35/39)	72.7% (88/121)	0.625	0.812	<0.001	0.738–0.886
		D2	79.5% (31/39)	86.0% (104/121)	0.654	0.827	<0.001	0.745–0.909
		D3	87.2% (34/39)	81.8% (99/121)	0.690	0.845	<0.001	0.772–0.918
		D4	87.2% (34/39)	83.5% (101/121)	0.707	0.853	<0.001	0.781–0.925
	Atypical, suspicious and positive cytology only	D1	92.1% (35/38)	22.5% (9/40)	0.146	0.573	0.267 *	0.446–0.700
		D2	81.6% (31/38)	60.0% (24/40)	0.416	0.708	0.002	0.591–0.825
		D3	89.5% (34/38)	47.5% (19/40)	0.370	0.685	0.005	0.566–0.804
		D4	89.5% (34/38)	52.5% (21/40)	0.420	0.710	0.001	0.593–0.826
Validation Study	All specimens	D2	82.6% (19/23)	84.5% (109/129)	0.671	0.836	<0.001	0.739–0.932
		D4	87.0% (20/23)	82.9% (107/129)	0.699	0.850	<0.001	0.761–0.938
	Atypical, suspicious and positive cytology only	D2	81.8% (18/22)	55.2% (16/29)	0.370	0.685	0.025	0.537–0.833
		D4	86.4% (19/22)	51.7% (15/29)	0.381	0.690	0.021	0.544–0.837

Abbreviations: D1–4, Definitions of chromosomal aneuploidy 1, 2, 3 and 4; AUC, Area under the ROC Curve. * Not significant for significance level *α* = 0.05. Accordingly, in this analysis it is not possible to discriminate between negative and positive follow-up results.

**Table 3:** Comparison of multi-color FISH results using definition D2 of chromosomal aneuploidy and either cytology or available information of a human stem cell transplantation in the clinical history. The data are reported in numbers.

Cutoff Study						
FISH	Cytology				HSCT	
	Negative	Atypical	Suspicious	Positive	No	Yes
FN	1	7	0	0	2	6
TN	80	22	2	0	34	70
FP	1	16	0	0	4	13
TP	0	15	8	8	13	18
Validation Study						
FISH	Cytology				HSCT	
	Negative	Atypical	Suspicious	Positive	No	Yes
FN	0	4	0	0	2	2
TN	93	15	1	0	33	77
FP	7	13	0	0	6	14
TP	1	7	4	7	7	12

Abbreviations: FISH, Fluorescence in situ hybridization; FN, false negative; TN, true negative; FP, false positive; TP, true positive; HSCT, hematopoietic stem cell transplantation.

**Table 4:** Analysis of a relative deletion with the 9p21 FISH assay. A cutoff of 16 or more cells with relative deletion was applied for the calculation of sensitivity and specificity because of the highest Youden Index.

		Cutoff	Sensitivity	Specificity	AUC	*p*-Value	AUC 5% Confidence Interval
Cutoff Study	All specimens	16	77.3%	63.9%	0.752	<0.001	0.662–0.842
	Atypical, suspicious and positive cytology only	16	55.3%	65.7%	0.587	0.200 *	0.455–0.719

Abbreviations: AUC, area under the curve; * Not significant for significance level *α* = 0.05.

## Data Availability

The data presented in this study are available in this article: “A New Multi-Color FISH Assay for Brush Biopsy-Based Detection of Chromosomal Aneuploidy in Oral (Pre)Cancer in Patients with Fanconi Anemia”.

## Appendix A

**Table A1:** One observers’ results of the multi-color FISH assay for possible cutoffs in the cutoff study with all specimens.

Definition	Cutoff-Point	Sensitivity	Specificity	Youden Index	Definition	Cutoff-Point	Sensitivity	Specificity	Youden Index
D1	1	0.974	0.438	0.412	D2	1	0.949	0.579	0.527
	2	0.974	0.587	0.561		2	0.923	0.686	0.609
	3	0.949	0.653	0.602		3	0.923	0.752	0.675
	4	0.923	0.686	0.609		4	0.872	0.810	0.682
	5	0.923	0.711	0.634		5	0.821	0.843	0.663
	6	0.897	0.727	0.625		6	0.795	0.860	0.654
	7	0.897	0.760	0.658		7	0.769	0.868	0.637
	8	0.897	0.769	0.666		8	0.692	0.884	0.577
	9	0.872	0.785	0.657		9	0.641	0.893	0.534
	10	0.846	0.785	0.631		10	0.641	0.901	0.542
	11	0.846	0.793	0.640		11	0.590	0.909	0.499
	12	0.795	0.802	0.597		12	0.564	0.917	0.481
	14	0.744	0.802	0.545		13	0.513	0.934	0.447
	15	0.718	0.818	0.536		15	0.487	0.950	0.438
	16	0.718	0.835	0.553		17	0.462	0.950	0.412
	18	0.718	0.843	0.561		20	0.436	0.950	0.386
	19	0.692	0.843	0.535		21	0.410	0.967	0.377
	20	0.667	0.860	0.526		23	0.333	0.967	0.300
	21	0.641	0.860	0.501		26	0.308	0.967	0.275
D3	1	0.974	0.529	0.503	D4	1	0.949	0.570	0.519
	2	0.949	0.636	0.585		2	0.949	0.686	0.635
	3	0.949	0.711	0.659		3	0.949	0.736	0.684
	4	0.923	0.752	0.675		4	0.897	0.785	0.683
	5	0.872	0.802	0.673		5	0.872	0.826	0.698
	6	0.872	0.818	0.690		6	0.872	0.835	0.707
	7	0.872	0.826	0.698		7	0.846	0.843	0.689
	8	0.846	0.826	0.673		8	0.744	0.843	0.587
	9	0.821	0.835	0.655		9	0.718	0.860	0.577
	10	0.769	0.835	0.604		10	0.692	0.876	0.568
	11	0.718	0.843	0.561		11	0.615	0.884	0.500
	12	0.667	0.843	0.510		12	0.590	0.901	0.491
	13	0.641	0.860	0.501		13	0.590	0.926	0.515
	14	0.641	0.868	0.509		14	0.538	0.926	0.464
	15	0.641	0.901	0.542		15	0.538	0.934	0.472
	16	0.590	0.909	0.499		16	0.538	0.950	0.489
	17	0.590	0.934	0.524		18	0.513	0.950	0.463
	19	0.564	0.942	0.506		21	0.487	0.967	0.454

**Table A2:** One observers’ results of the multi-color FISH assay for possible cutoffs in the cutoff study calculated for those samples with atypical, suspicious and positive cytology only.

Definition	Cutoff-Point	Sensitivity	Specificity	Youden Index	Definition	Cutoff-Point	Sensitivity	Specificity	Youden Index
D1	1	1.000	0.025	0.025	D2	1	0.974	0.150	0.124
	3	0.974	0.075	0.049		2	0.947	0.250	0.197
	4	0.947	0.150	0.097		3	0.947	0.350	0.297
	5	0.947	0.175	0.122		4	0.895	0.500	0.395
	6	0.921	0.225	0.146		5	0.842	0.550	0.392
	7	0.921	0.300	0.221		6	0.816	0.600	0.416
	8	0.921	0.325	0.246		7	0.789	0.625	0.414
	9	0.895	0.375	0.270		8	0.711	0.675	0.386
	10	0.868	0.375	0.243		9	0.658	0.700	0.358
	11	0.868	0.400	0.268		11	0.605	0.725	0.330
	12	0.816	0.425	0.241		12	0.579	0.750	0.329
	14	0.763	0.425	0.188		13	0.526	0.800	0.326
	15	0.737	0.475	0.212		15	0.500	0.850	0.350
	16	0.737	0.500	0.237		17	0.474	0.850	0.324
	18	0.737	0.525	0.262		20	0.447	0.850	0.297
	19	0.711	0.525	0.236		21	0.421	0.900	0.321
	20	0.684	0.575	0.259		23	0.342	0.900	0.242
	21	0.658	0.575	0.233		26	0.316	0.900	0.216
	23	0.658	0.625	0.283		28	0.289	0.950	0.239
	24	0.658	0.675	0.333		30	0.263	0.950	0.213
	25	0.658	0.700	0.358		32	0.211	0.975	0.186
	26	0.632	0.750	0.382		34	0.184	0.975	0.159
D3	1	1.000	0.075	0.075	D4	1	0.974	0.125	0.099
	2	0.974	0.125	0.099		2	0.974	0.250	0.224
	3	0.974	0.250	0.224		3	0.974	0.325	0.299
	4	0.947	0.350	0.297		4	0.921	0.450	0.371
	5	0.895	0.425	0.320		5	0.895	0.500	0.395
	6	0.895	0.475	0.370		6	0.895	0.525	0.420
	7	0.895	0.500	0.395		7	0.868	0.550	0.418
	8	0.868	0.500	0.368		8	0.763	0.550	0.313
	9	0.842	0.525	0.367		9	0.737	0.600	0.337
	10	0.789	0.525	0.314		10	0.711	0.650	0.361
	11	0.737	0.550	0.287		11	0.632	0.650	0.282
	12	0.684	0.550	0.234		12	0.605	0.700	0.305
	13	0.658	0.575	0.233		13	0.605	0.775	0.380
	14	0.658	0.600	0.258		14	0.553	0.775	0.328
	15	0.658	0.700	0.358		15	0.553	0.800	0.353
	16	0.605	0.725	0.330		16	0.553	0.850	0.403
	17	0.605	0.800	0.405		18	0.526	0.850	0.376
	19	0.579	0.825	0.404		21	0.500	0.900	0.400

## Appendix B

**Table A3:** Enhanced follow-up data for 24 lesions from 21 FA patients with false-positive FISH results using the definition D2 of chromosomal aneuploidy.

**Enhanced Follow-Up (Range 2–5 Years) without HG OED or SCC**		
**FP FISH**	**Histology**	
**Oral Region**	**Result**	**Time Interval ***
Gingiva	---	---
Gingiva	---	---
Gingiva	---	---
Gingiva	---	---
Gingiva	---	---
Cheek	---	---
Tongue	---	---
Tongue	---	---
Tongue	---	---
Gingiva	---	---
**HG OED or SCC Somewhere in the Oral Cavity but not in the Vicinity of the Brushed Lesion**		
**FP FISH**	**Histology**	
**Oral Region**	**Result**	**Time Interval**
Gingiva	SCC	NR
Gingiva	SCC,	<1 year
	HG OED	<1 year
	SCC,	2 Years
Tongue	HG OED	NR
Gingiva	SCC	NR
Tongue	SCC	NR
HG OED or SCC in the vicinity of oral brushing or at the same place in the clinical course		
FP FISH	Histology	
Oral Region	Result	Time Interval
Gingiva	SCC,	<1 year
Gingiva	HG OED	NR
Gingiva	HG OED	NR
Gingiva	SCC	<1 year
Cheek	SCC	2 Years
Tongue	HG OED	1 Year
Lip	SCC	NR
Cheek	SCC	2 Years
Gingiva	SCC	NR

Abbreviations: FISH, Fluorescence in situ hybridization; FP, false-positive; SCC, squamous cells carcinoma; HG OED, high-grade oral epithelial dysplasia; NR, not reported.
